# Key Insights into Gut Alterations in Metabolic Syndrome

**DOI:** 10.3390/jcm14082678

**Published:** 2025-04-14

**Authors:** Adrian Boicean, Cristian Ichim, Sabina-Maria Sasu, Samuel Bogdan Todor

**Affiliations:** Faculty of Medicine, Lucian Blaga University of Sibiu, 550169 Sibiu, Romania; adrian.boicean@ulbsibiu.ro (A.B.); samuelbogdant@gmail.com (S.B.T.)

**Keywords:** gut, dyslipidemia, obesity, hypertension, FMT

## Abstract

Over time, extensive research has underscored the pivotal role of gut microbiota in the onset and progression of various diseases, with a particular focus on fecal microbiota transplantation (FMT) as a potential therapeutic approach. The practice of transferring fecal matter from a healthy donor to a patient provides valuable insights into how alterations in gut microbiota can impact disease development and how rectifying dysbiosis may offer therapeutic benefits. Re-establishing a balanced symbiotic relationship in the gastrointestinal tract has shown positive results in managing both intestinal and systemic conditions. Currently, one of the most pressing global health issues is metabolic syndrome—a cluster of conditions that includes insulin resistance, lipid imbalances, central obesity and hypertension. In this context, FMT has emerged as a promising strategy for addressing key components of metabolic syndrome, such as improving insulin sensitivity, body weight and lipid profiles. However, further well-structured studies are needed to refine treatment protocols and establish the long-term safety and efficacy of this intervention.

## 1. Introduction

It is undeniable that metabolic abnormalities have reached epidemic levels, and this ongoing trend presents a significant challenge for clinicians worldwide, as incidence rates continue to rise without showing any signs of slowing down [1,2,3,4]. Investigations into the underlying mechanisms of metabolic syndrome have increasingly pointed to the gut microbiota as a pivotal contributor to its pathophysiology [5]. The intricate and dynamic nature of the intestinal microbiota initiates a cascade of physiological processes, with disruptions in its composition—known as dysbiosis—adversely impacting overall health. This has led to the description of the microbiota as an essential component within the host, reflecting its extensive and vital roles, as well as the intricate chain reactions it can initiate when dysregulated [6]. Comprising trillions of microorganisms, this highly diverse and adaptable ecosystem has garnered growing attention in recent medical research. Its modulation has become the target of numerous therapeutic interventions aimed at restoring microbial equilibrium and, consequently, improving health outcomes [7,8]. Since each component of metabolic syndrome has been associated with alterations in gut flora, researchers have begun exploring whether intentionally manipulating this community could counteract the syndrome’s detrimental effects. In this context, fecal microbiota transplantation (FMT)—initially developed as a treatment for recurrent Clostridioides difficile infection—has gained prominence as a novel therapeutic approach for addressing various manifestations of metabolic syndrome [9]. Epidemiological evidence further underscores the seriousness of metabolic syndrome, linking it to a significantly elevated risk of cardiovascular disease, type 2 diabetes and several types of cancer. Individuals diagnosed with metabolic syndrome face a fivefold increase in the likelihood of developing type 2 diabetes and are three times more likely to suffer from cardiovascular conditions. Data from large cohort studies, including a U.S.-population-based analysis involving 900,000 individuals, indicate that obesity accounts for approximately 14% of cancer-related deaths in men and 20% in women. Additionally, dyslipidemia—particularly characterized by low HDL cholesterol and elevated LDL cholesterol—has been associated with malignancies of the lung, colon, stomach, breast and hematopoietic system [9]. Altered gut microbiota profiles in individuals with metabolic syndrome are marked by decreased microbial diversity and functional changes that promote excessive energy storage and chronic inflammation. Research involving obese twin pairs has demonstrated a reduced abundance of Bacteroidetes and Actinobacteria, with relatively stable levels of Firmicutes. These findings suggest that metabolic disturbances are likely driven by functional microbial imbalances rather than simple fluctuations in diversity. Notably, dysbiosis in metabolic syndrome features an increased presence of Proteobacteria and Bacteroidetes, diminished populations of beneficial species such as Akkermansia muciniphila, and a rise in pathogenic microorganisms like Campylobacter and Shigella [5]. Emerging data indicate that FMT holds potential not only for reestablishing a balanced intestinal microbiota but also for alleviating key features of metabolic syndrome, including obesity, insulin resistance, dyslipidemia and hypertension. This positions FMT as a promising integrative approach to managing this complex and multifaceted condition. The aim of this review is to gain a deeper understanding of the specific microbial alterations associated with each component of metabolic syndrome, which will be essential to fully unlock the therapeutic potential of this intervention.

## 2. Obesity-Related Shifts in Gut Microbial Communities

Research has demonstrated clear parallels in the gut microbiota profiles of related individuals, highlighting a genetic predisposition toward obesity. More importantly, these studies reveal that obesity is often accompanied by a marked reduction in microbial diversity within the gut and shifts in specific bacterial populations. For instance, the work of Turnbaugh et al., using 16S rRNA sequencing, identified a decrease in Bacteroidetes and an increase in Actinobacteria in obese subjects compared to their lean counterparts. Furthermore, metabolic pathway analysis in these individuals revealed enrichment in carbohydrate metabolism pathways, such as phosphotransferase systems (PTSs), with statistical confirmation provided by bootstrap analysis. Supporting this link, experiments in germ-free mice colonized with microbiota from obese donors resulted in significant weight gain and increased adiposity [10]. Microbial diversity has been further characterized by Le Chatelier et al., who quantified bacterial variety through gene count analysis. Low gene count profiles, often associated with conditions like inflammatory bowel disease, obesity and chronic inflammation in elderly individuals, are dominated by microbial species such as Bacteroides, Ruminococcus, Campylobacter and Staphylococcus. Conversely, high gene count profiles are associated with beneficial species like Faecalibacterium, Bifidobacterium, Akkermansia and Methanobrevibacter [11]. Two key bacterial groups, Firmicutes and Bacteroidetes, have long been studied as potential indicators of obesity-related dysbiosis in both children and adults. While shifts in their relative abundance were initially considered strong markers, variations between individuals—even within the same population—and influences from diet, environment and antibiotic use have hindered the acceptance of this ratio as a definitive diagnostic tool [12,13]. Despite numerous studies following the pioneering work by Ley et al., which suggested an imbalance in these two phyla in obesity, results remain inconclusive and inconsistent across studies [14]. The impact of the allogenic microbiome has also been explored through elegant experimental models. In one notable study, germ-free mice colonized with microbiota from twin donors—one obese and one lean—exhibited distinct metabolic outcomes. Mice receiving microbiota from the obese twin displayed increased fat deposition, along with higher Firmicutes and reduced Bacteroidetes, while those colonized with microbiota from the lean twin maintained a lean phenotype. Additionally, when obese and lean mice were cohoused, the obese mice experienced a reduction in weight gain, explained by the horizontal transfer of beneficial microbiota through coprophagy [15].

Dietary patterns exert a significant influence on gut microbial composition, as demonstrated by David et al. Their study examined the effects of animal-based versus plant-based diets. Diets rich in animal products favored the proliferation of bile-tolerant bacteria such as Alistipes, Bilophila and Bacteroides, whereas fiber-degrading bacteria like Prevotella thrived on plant-based diets. Notably, returning to a habitual diet allowed the microbiota to revert to its baseline composition. These dietary-induced changes were also reflected at the level of gene expression, potentially accounting for metabolic variations linked to diet [16]. Consequently, alterations in the gut microbiota associated with obesity occur at both microbial and enzymatic levels, with genetics and dietary habits playing critical, intertwined roles in shaping these outcomes.

## 3. Microbiota-Derived Metabolites and Inflammatory Pathways in Hypertension

The relationship between elevated blood pressure and gut microbiota composition has garnered significant attention in recent years. Li et al. provided compelling evidence that both hypertension and prehypertension are closely associated with alterations in the gut microbial community [17]. Similar to the observations made by Le Chatelier et al. regarding reduced microbial gene count and its connection to various diseases, Li et al. emphasized the correlation between diminished microbial diversity and the presence of prehypertension and hypertension [11,17]. Moreover, certain bacterial genera, notably Prevotella and Klebsiella, have been strongly linked to these conditions, with Prevotella being particularly associated with elevated levels of stearic acid—a metabolite implicated in hypertension pathogenesis.

Beyond taxonomic shifts, functional changes in the gut microbiota of hypertensive individuals have also been noted. These include upregulation of several metabolic and transport pathways, such as the phosphotransferase system (PTS), bacterial secretion systems and processes involved in phospholipid transport and phosphatidylethanolamine biosynthesis [17]. Of particular concern is the role of Gram-negative bacteria and their cell wall component, lipopolysaccharide (LPS). Overgrowth of bacteria such as Prevotella and Klebsiella leads to increased LPS release, which subsequently binds to Toll-like receptor 4 (TLR4) on immune cells. This interaction initiates a signaling cascade resulting in the activation of the nuclear factor kappa B (NF-κB) pathway and the production of pro-inflammatory cytokines, including tumor necrosis factor-alpha (TNF-α), interleukin-1 beta (IL-1β) and interleukin-6 (IL-6) [18,19,20]. Persistent activation of this inflammatory pathway promotes chronic low-grade inflammation, a key contributor to the development and progression of hypertension [21,22,23]. Another critical factor in blood pressure regulation is the activity of short-chain fatty acids (SCFAs), such as acetate, propionate and butyrate. These compounds are fermentation products of dietary fibers in the colon—a process reliant on the gut microbiota, given the absence of human digestive enzymes capable of breaking down these fibers. SCFAs exert antihypertensive effects through multiple mechanisms: they modulate inflammation, influence immune responses and impact renal physiology, including the regulation of renin secretion. Additionally, SCFAs have a direct vasodilatory effect by acting on G-protein-coupled receptors (GPCR41 and GPCR43), contributing to blood pressure reduction [24,25,26,27,28,29]. Experimental studies aimed at evaluating antihypertensive interventions have demonstrated that each of these SCFAs—acetate, butyrate and propionate—plays a distinct role in normalizing blood pressure values [30,31,32,33].

## 4. Intestinal Microbiota and Its Impact on Lipid Homeostasis

Dyslipidemia, marked by abnormal levels of cholesterol and triglycerides, often coexists with disturbances in lipoprotein metabolism [34]. Emerging research suggests that the gut microbiota plays a pivotal role in the pathogenesis of this condition through several distinct mechanisms. One key process involves the conversion of dietary choline into trimethylamine, which, upon hepatic oxidation, forms trimethylamine N-oxide (TMAO) [35]. TMAO has garnered attention as a potential independent marker for cardiovascular risk [36,37], with evidence linking elevated levels to both cholesterol imbalances and the promotion of vascular inflammation, which can exacerbate dyslipidemia [38,39]. In addition to TMAO production, short-chain fatty acids (SCFAs) produced by gut microbes have significant effects on lipid metabolism. Butyrate, for instance, helps regulate fat storage by inhibiting triglyceride synthesis through reduced lipase activity. Propionate stimulates the release of glucagon-like peptide-1 (GLP-1), which not only enhances insulin secretion but also promotes lipogenesis while inhibiting lipolysis. Furthermore, elevated acetate levels can increase insulin secretion and stimulate appetite via the parasympathetic nervous system [40,41,42]. Moreover, bile acids, which are primarily responsible for the elimination of excess cholesterol, are also metabolized by the gut microbiota. Disruptions in the gut microbiota can lead to changes in bile acid metabolism, influencing health outcomes, weight gain and lipid accumulation [43,44].

## 5. The Gut Microbiota’s Influence on Insulin Resistance

The link between insulin resistance and the gut microbiota is governed by a complex array of biological processes. A central player in this interaction is the production of short-chain fatty acids, which are essential for maintaining metabolic balance in the host. The release of critical intestinal hormones such as peptide YY and glucagon-like peptide-1 (GLP-1) relies on the presence of SCFAs, which activate free fatty acid receptors GPR41 and GPR43. The activation of these receptors promotes satiety, enhances insulin sensitivity and supports glucose tolerance. Disruptions in microbial composition that lead to decreased SCFA production can impair these metabolic processes, thereby fostering the onset of insulin resistance [45,46,47]. Metabolic endotoxemia represents another key factor, marked by the entry of lipopolysaccharides (LPSs) into the bloodstream, typically following a high-fat, calorie-rich meal. LPS translocation activates Toll-like receptor 4 (TLR4), triggering an inflammatory cascade that interferes with insulin signaling, thus contributing to metabolic dysfunction. This process is aggravated by an increase in intestinal permeability, often described as “leaky gut”, which allows LPSs to more easily enter the circulatory system, further promoting systemic inflammation [48,49]. In animal models, studies have shown that continuous LPS infusion or a high-fat diet leads to weight gain, increased adiposity and the onset of chronic low-grade inflammation [50]. Similarly, in human studies, Agwunobi et al. observed a two-phase response to LPS infusion: an initial surge in glucose uptake, likely driven by cytokines from the inflammatory response, followed by the development of insulin resistance [51]. In conclusion, LPS, as a product of gut microbial activity, plays a central role in triggering inflammatory responses that disrupt insulin signaling. As such, imbalances in gut microbiota composition and the presence of endotoxemia are significant contributors to the development of insulin resistance and the chronic inflammation associated with metabolic disturbances.

The key findings regarding gut changes in metabolic syndrome are summarized in the studies presented in Table 1.

## 6. Fecal Microbiota Transplantation Therapy in Metabolic Syndrome

FMT is a therapeutic approach where fecal material from a healthy donor is introduced into the gastrointestinal tract of a patient to correct dysbiosis and restore balance to the gut microbiota. This treatment has shown notable success in managing recurrent Clostridioides difficile infections [9,52,53]. Following these positive outcomes, researchers began to explore whether FMT could also benefit other intestinal or systemic conditions where the gut microbiota is implicated in disease pathophysiology. As a result, FMT is currently being studied for its potential effects on inflammatory bowel disease, irritable bowel syndrome, obesity, metabolic syndrome, autoimmune disorders, neuropsychiatric conditions influenced by the gut–brain axis, as well as pancreatic and liver diseases [54,55,56,57,58,59,60,61,62]. To ensure the safety and efficacy of FMT, extensive screening is conducted on the donor, including medical history review, physical examination, serological testing and stool analysis. Similarly, recipients undergo testing for infectious diseases, including Human Immunodeficiency Virus (HIV), syphilis and hepatitis B and C. While there are exceptional cases where FMT may be performed without screening, such situations are avoided whenever possible [63]. There are various methods for administering processed fecal matter, including nasoenteric tubes, upper endoscopy, oral capsules, colonoscopy and retention enema, each offering distinct advantages and drawbacks [64]. Due to the potential risks associated with FMT, advancements have been made in processing techniques. These now include not only saline dilution and filtration but also microfiltration and repeated centrifugation, a process known as Washed Microbiota Transplantation (WMT). A study by Zhang et al. utilizing advanced sequencing, spectrometry, metabolomics and statistical analysis demonstrated that WMT offers greater precision and safety compared to traditional FMT by implementing stringent quality controls, reducing adverse effects for recipients [65]. Fecal microbiota transplantation has gained attention for its potential therapeutic effects in treating metabolic syndrome, targeting conditions such as insulin resistance, obesity and associated metabolic disturbances. Insulin resistance, a key factor in the development of obesity and related disorders, has become a primary focus for FMT interventions, particularly after studies demonstrated improved insulin sensitivity following microbiota transfer.

Wu et al. conducted a study comparing three groups: FMT alone, FMT with metformin and metformin alone. After 4 weeks, significant improvements were noted in insulin resistance, body mass index (BMI), hemoglobin A1c and blood glucose levels in both FMT groups, whether or not metformin was included [66]. Similarly, in a study by Ng et al., improvements in lipid profiles and liver stiffness were observed in both obese patients and those with type 2 diabetes, particularly when FMT was combined with lifestyle modifications. This study also found successful engraftment of donor microbiota with a noticeable increase in Bifidobacterium and Lactobacillus populations [67]. In a separate study, Su et al. demonstrated that combining FMT with dietary changes accelerated weight loss and positively impacted fasting blood glucose, blood pressure and lipid profiles, with a shift in microbiota from Bacteroides to Prevotella dominance [68]. Additionally, the incorporation of low-fermentable microcrystalline cellulose fiber after FMT was shown to enhance insulin sensitivity by modulating interactions between microbiota and the gut lumen [69]. These findings suggest that FMT is most effective when integrated with other established therapeutic strategies. Furthermore, a predictive pattern for favorable outcomes in patients with type 2 diabetes was observed by analyzing pre-treatment fecal samples. Those with elevated Rikenellaceae and Anaerotruncus levels showed greater reductions in blood glucose, hemoglobin A1c and uric acid after treatment, indicating that microbiota composition prior to treatment may help predict clinical response [70].

However, not all studies have produced consistent results. For example, interventions in obese patients undergoing bariatric surgery or in adolescents showed no substantial changes in metabolic markers [71,72]. Despite the lack of significant changes in metabolic profiles, Leong et al. did observe a reduction in the android-to-gynoid fat ratio in adolescent females post-FMT [71]. Furthermore, laparoscopic sleeve gastrectomy and Roux-en-Y gastric bypass did not result in notable improvements when combined with FMT [72]. In successful FMT engraftments, the recipient’s microbiota often mirrors that of the donor, particularly in the presence of key species like Faecalibacterium, which is involved in butyrate production and bile acid hydrolysis. As shown by Vrieze et al., FMT can significantly increase butyrate-producing bacteria, such as Roseburia intestinalis and Eubacterium hallii, both of which contribute to improvements in insulin sensitivity [73,74,75]. Conversely, a reduction in butyrate-producing bacteria is linked to higher intestinal permeability and bacterial translocation, which can exacerbate systemic inflammation and metabolic dysregulation [76,77]. Surprisingly, the study by Kootte et al. found a reduced presence of Roseburia in responders to autologous FMT, even though insulin sensitivity improved, which contradicts earlier reports. Other beneficial bacteria, such as Akkermansia muciniphila and Eubacterium, were also prevalent in the responders’ microbiota [78]. A long-term study on the effects of FMT on metabolic syndrome found that microbiota diversity remained high for up to 12 months post-transplant, but no significant biochemical or anthropometric changes were observed, likely due to the loss of anaerobic species, such as Fecalibacterium prausnitzii and Akkermansia muciniphila, during the transfer process [79].

Dietary factors also play a role in FMT’s effectiveness. In a study by Smits et al., no changes in trimethylamine (TMA) or trimethylamine-N-oxide (TMAO) levels were seen after autologous FMT from a vegan donor. However, certain butyrate-producing bacteria, such as Lachnospira bovis and Anaerostipes, were abundant in the feces of the vegan donor, along with bacteria from the Clostridium genus, which is linked to metabolic syndrome [80].

Regarding the route of administration, Zhong et al. demonstrated that Washed Microbiota Transplantation via the transendoscopic enteral route led to more pronounced effects, particularly in hypotensive patients who had not previously been treated with antihypertensive medications. The transplant had no significant effect on blood pressure in patients with normal baseline levels [81]. Retrospective studies by Wu et al. have highlighted the short-, medium- and long-term benefits of WMT, showing reductions in fasting blood glucose, improved lipid profiles and decreases in blood pressure, all contributing to a reduced risk of atherosclerotic cardiovascular disease in high-risk individuals [82,83].

## 7. Alternative Approaches for Modulating the Gut Microbiome

The treatment of metabolic syndrome combines pharmacological and non-pharmacological strategies to prevent type 2 diabetes and reduce cardiovascular risk, with bariatric surgery often used to decrease body mass index. In addition to fecal microbiota transplantation, several alternative methods for modulating the gut microbiome are being investigated as potential therapeutic options for metabolic syndrome.

### 7.1. Microbiome-Targeted Interventions: From Probiotics to Polyphenols

Alternative strategies for modulating the microbiota include probiotics, prebiotics, synbiotics, postbiotics, microbiome-targeted diets, short-chain fatty acid (SCFA) supplementation, bacteriophage therapy and polyphenol supplementation [84,85,86,87,88,89,90,91,92]. In their systematic review of randomized clinical trials, Tenorio-Jiménez et al. found modest improvements in components of metabolic syndrome with the use of various probiotics, such as Lactobacillus plantarum and Bifidobacterium lactis [84,93,94]. For instance, Lactobacillus plantarum, when consumed through fermented milk, resulted in significant reductions in glucose and homocysteine levels in postmenopausal women, compared to those who consumed non-fermented milk [93]. Similarly, Bifidobacterium lactis led to reductions in total cholesterol, glucose and interleukin-6 (a cytokine linked to obesity) [94]. Probiotics, which are beneficial bacteria found in fermented foods, differ from prebiotics—indigestible fibers that promote fermentation in the gut and the production of short-chain fatty acids. O’Connor et al. reviewed studies on prebiotics, including resistant starches, inulin and fructo-oligosaccharides, and found them to positively affect body weight, inflammation, glucose metabolism, dyslipidemia and hypertension in patients with metabolic disorders [85]. Synbiotics, a combination of both probiotics and prebiotics, have shown promise in reducing cardiovascular risk and improving anthropometric measurements [86]. Postbiotics, which include a wide range of compounds produced by probiotics, have been found to play diverse roles in correcting dysbiosis and metabolic abnormalities. These roles include anti-obesogenic effects, antioxidant properties and the regulation of glucose metabolism and lipid profiles [87].

### 7.2. Personalized Diets

A personalized diet can induce metabolic changes, such as modifications in postprandial glycemic responses. However, the effects of different dietary plans vary between individuals due to their unique intestinal microbiota profiles, which influence how they respond to the same types of food. For instance, individuals with a higher Prevotella-to-Klebsiella ratio may experience greater weight loss on a high-fiber diet compared to those with a lower ratio of these bacteria [88]. In relation to short-chain fatty acids, acetate serves as an example. When its production is induced in the body, such as through prebiotic administration, it can enhance insulin sensitivity. Furthermore, direct oral administration of acetate, in the form of acetic acid (vinegar), can help normalize blood glucose levels [89]. Foods also contain polyphenols, which are being studied for their potential in combating components of metabolic syndrome. Resveratrol, for example, is being investigated for its role in weight loss, grape seeds for reducing blood pressure and quercetin for lowering lipids and serum glucose levels [91,95].

### 7.3. Fecal Virome Transplantation

A novel concept in the scientific community is the potential to replace fecal microbiota transplantation with fecal virome transplantation (FVT), where filtered, sterile donor fecal matter—free of bacteria—is used to administer treatment mediated by bacteriophages [90]. A mouse study has shown that FVT alleviated symptoms of obesity and type 2 diabetes [96]. Modern treatments have shown the spectacular benefits of phage therapy, which were overshadowed by the emergence of antibiotics, but are now of great importance, especially due to antibiotic resistance. Numerous studies have demonstrated the ability of phages to modulate the intestinal microbiota, proving to be a valuable tool in the treatment of pathologies related to metabolic syndrome and beyond [97,98,99,100].

### 7.4. Antibiotics

Antibiotic therapy, both targeted and broad-spectrum, is often used to modulate the microbiota due to its ability to destroy harmful bacteria and restore balance within the body. However, the selection of antibiotic therapy must be carried out carefully and should provide benefits to the patient rather than risks. Many reactions to certain antibiotics are well known, and they can even lead to the undesired effect of exacerbating dysbiosis, favoring the growth of unwanted bacteria and generating aggressive infections—e.g., *Clostridioides difficile* infection occurring post-antibiotic therapy [101,102,103,104].

### 7.5. Genetic Engineering

Current medicine focuses heavily on metabolic engineering, which has recently been applied to develop therapeutic and diagnostic strategies targeted at the intestinal microbiota, including next-generation (genetically modified) probiotics and synthetic microbial consortia with specific functions. Certainly, genetic engineering has its current limitations and could even cause more harm than good, but significant progress is being made in this field. To enhance efficiency, computational models and genetic tools are used to manipulate microorganisms. Engineered microbes could help in efficiently modulating the microbiota, precisely because they can be designed to target specific problems and resolve them in an efficient manner [105,106,107,108,109].

### 7.6. Bile Acid Modulation

Bile acids regulate the gut microbiota through their direct antimicrobial activity and interactions with nuclear and membrane receptors, thereby influencing intestinal homeostasis and immune responses. The complex relationship between bile acids and the microbiota plays a key role in maintaining the intestinal barrier, host immunity and resistance to enteric pathogens. Imbalances in bile acid metabolism can disrupt intestinal physiology and promote the expansion of harmful bacterial strains. Nevertheless, future studies may reveal the therapeutic potential of modulating bile acids as a means to restore microbiota balance, potentially offering fewer risks and adverse effects compared to other available approaches [110,111,112].

Despite the growing potential of these therapies, patient perceptions of these treatments can vary. While some patients view FMT as an innovative solution to their symptoms, others may find the concept unappealing or even repugnant, especially when it involves the idea of ingesting fecal matter. In these cases, colonoscopy is generally preferred as the route for administration over ingestion [98]. When comparing the different methods, it is important to note that postbiotics and bacteriophage therapy are still in the early stages of clinical application and FVT is strain-specific. Probiotics, prebiotics and synbiotics have demonstrated positive effects on insulin sensitivity, inflammation, SCFA production and other metabolic parameters, but they show fluctuating results due to individual variability in responses. Additionally, their effects can be dose-dependent and strain-specific. Microbiome-targeted diets lead to sustained metabolic improvements but require long-term patient adherence. SCFA supplementation is an effective approach, particularly when combined with other therapies like probiotics. However, polyphenol supplementation faces challenges related to bioavailability. While FMT is a promising method for rapidly establishing intestinal microbial colonization, it remains donor-dependent and carries inherent risks, despite rigorous donor screening processes [113,114,115,116,117,118,119,120,121,122,123,124].

## 8. Risks and Potential Ethical Implications of Fecal Microbiota Transplantation

Overall, fecal microbiota transplantation has been shown to be safe, as demonstrated by the majority of studies. Adverse effects are generally minor, including abdominal pain, bowel movement disturbances and complications related to the method of administration. However, FMT is approved in most countries only as a last-resort treatment for Clostridioides difficile infections. Its use in metabolic syndrome or other pathologies remains largely unregulated, and physicians should proceed with caution when considering this form of therapy, especially since the FDA issued a warning following a patient’s death after undergoing FMT. Despite this, ethics committees in various institutions have approved its experimental use in clinical trials. Significant progress is being made, with an increasing number of valuable insights being added to the scientific literature [125,126,127,128].

## 9. Conclusions

Given the immense diversity of bacteria within the gut, it is crucial to recognize the intricate web of interactions that occur. Each individual harbors a unique microbiota profile, which plays a key role in determining how they respond to treatment. While numerous factors—such as the donor, recipient and underlying disease—affect the outcome, all evidence suggests that metabolic syndrome is significantly influenced by the gut microbiome. Refining therapeutic approaches, such as Washed Microbiota Transplantation, which reduces side effects, may allow these microbiota transplants to become a viable and reliable treatment option. Moving forward, focusing on the gut microbiota offers a promising strategy for managing metabolic disorders. Fecal Microbiota Transplantation shows potential in improving insulin sensitivity, though further research is necessary to fully understand its efficacy. Personalized therapies, including probiotics and customized dietary interventions, could enhance patient outcomes. Future studies should aim to optimize these approaches and explore how they can be effectively integrated with traditional treatments.

## Figures and Tables

**Table 1:** The main findings related to gut alterations in metabolic syndrome.

References	Primary Observations	Gut Microbiota Changes
[10]	Obese individuals show alterations in gut microbiota composition compared to lean individuals.	Decrease in Bacteroidetes, increase in Actinobacteria; enriched carbohydrate metabolic pathways.
[11]	Gene count in gut microbiota correlates with metabolic conditions such as obesity and inflammation.	Low gene count: Bacteroides, Ruminococcus, Campylobacter, Staphylococcus; High gene count: Faecalibacterium, Bifidobacterium, Akkermansia, Methanobrevibacter.
[14]	The Firmicutes/Bacteroidetes ratio differs in obese vs. lean individuals.	Increase in Firmicutes, decrease in Bacteroidetes in obesity.
[15]	Microbiota transfer from obese to germ-free mice induces weight gain.	Increased Firmicutes, reduced Bacteroidetes in obese phenotype.
[16]	Diet modulates gut microbiota composition within days.	Animal-based diet: Increase in Alistipes, Bilophila, Bacteroides; plant-based diet: increase in Prevotella.
[17]	Gut microbial diversity is reduced in hypertension.	Increased Prevotella and Klebsiella; functional enrichment of phosphotransferase system (PTS).
[18]	LPS-induced inflammation leads to hypertension through immune response activation.	Increased abundance of Gram-negative bacteria producing LPS.
[33]	Increases in circulating and fecal butyrate are associated with reduced blood pressure and hypertension.	Increased butyrate in feces.
[34]	The gut microbiome is involved in the conversion of choline to TMAO, influencing plasma cholesterol levels and cardiovascular risk.	Increased TMAO production due to microbiota conversion of choline.
[44]	Dysbiosis affects bile acid metabolism, leading to weight gain and lipid accumulation.	Microbial imbalance leading to alterations in bile acid metabolism.
